# Mechanical Thrombectomy for Ischaemic Stroke: The First UK Case Series

**DOI:** 10.1371/journal.pone.0082218

**Published:** 2013-12-26

**Authors:** Nasar Ahmad, Sanjeev Nayak, Changez Jadun, Indira Natarajan, Palbha Jain, Christine Roffe

**Affiliations:** 1 University Hospital of North Staffordshire, Stoke-on-Trent, United Kingdom; 2 Department of Interventional Neuroradiology, University Hospital of North Staffordshire, Stoke-on-Trent, United Kingdom; 3 School of Medicine, Keele University, Keele, United Kingdom; 4 Institute for Science and Technology in Medicine, Keele University, Keele, United Kingdom; 5 North Staffordshire Combined Healthcare Trust, Stoke-on-Trent, United Kingdom; University of Münster, Germany

## Abstract

**Background and Purpose:**

Endovascular treatments have the potential to accelerate reperfusion in acute ischaemic stroke with large vessel occlusion. In the UK only a few stroke centres offer this interventional option. The University Hospital of North Staffordshire (UHNS) has treated the largest number of cases in the UK. Results of the first 106 endovascular treatments (EVT) are presented here.

**Methods:**

All patients treated with EVT (intra-arterial thrombolysis (IAT), mechanical thrombectomy (MT) or both, or an attempt at intervention) for acute stroke at UHNS, Stoke-on-Trent, UK, were entered into a prospective register. Baseline demographic and clinical data, the National Institutes for Health Stroke Scale (NIHSS), imaging results including Thrombolysis in Cerebral Infarction (TICI) score, and complications were recorded. Mortality, and modified Rankin score (mRS) were assessed at 90 days.

**Results:**

From December 2009 to January 2013 106 patients (mean age 64 years, median baseline NIHSS 18) were treated with EVT (thrombectomy ± IAT 83%, IAT alone 13%, neither 4%). Seventy-eight per cent of occlusions were in the anterior circulation. Intravenous bridging thrombolysis was performed in 81%. Revascularization was successful (TICI 2b/3) in 84%. The median time from stroke onset to the end of the procedure was 6 h 03 min. A good outcome (mRS≤2) at 90 days was achieved in 48% with a mortality of 15%. Fatal or nonfatal symptomatic intracranial haemorrhage (sICH) within 10 days occurred in 9%. The median length of stay was 14 days (31% discharged home ≤7 days).

**Conclusions:**

EVT led to good clinical outcomes in almost 50% of patients with severe strokes.

## Introduction

A key determinant of good recovery from acute stroke is early restoration of blood flow to the ischaemic brain [1]. Intravenous thrombolysis (IVT) with alteplase has made a great difference to stroke treatment worldwide, and is now used in 8% of UK stroke patients [2]. However, recanalization rates with IVT are poor in patients with occlusion of a large intracerebral artery (30% in basilar artery occlusions, 30% in proximal middle cerebral artery occlusions and 6% in terminal internal carotid artery occlusions) [3]. Endovascular treatment (EVT) applies thrombolytic agents directly into the thrombus (IAT) or mechanically extracts the clot (MT). A meta-analysis of 53 studies including a total of 2066 strokes showed considerably higher recanalization rates with EVT (46% for IVT, 63% for IAT, 68%, for combined IV/IA, and 84% for MT). Early recanalization was associated with a 5-fold increase in the proportion of patients who were alive and independent at 3 months [1]. 

MT is considered experimental in the UK [4], although open studies strongly suggest a role for EVT for acute ischemic stroke. However, three recently published randomized controlled trials (RCTs) [5-7] did not show benefit, and further RCTs using tighter inclusion criteria and more effective devices are progressing. A UK-based consensus statement drawn up by bodies representing neuroradiologists, the British Association of Stroke Physicians and the Department of Health, outlined service requirements for provision of EVT in 2012 [8]. The National Stroke Strategy for England includes the plan to provide access to acute neurointerventional treatments for all stroke patients. A national survey in 2010 showed that 23 stroke centres in the UK had at least some experience with providing interventional treatments for acute stroke, and that 6 centres did one or more interventions per month [9]. An editorial in the British Medical Journal in 2012 suggested that interventional treatments will probably be the treatment of choice for patients with an ischaemic stroke due to large vessel occlusion, but that more research is needed [10]. 

It is important that active centres share their experience by making their data available for scrutiny. There is as yet no published case series for the United Kingdom. In this paper we present our local protocol for EVT and the results of the first 106 cases. We now aim to expand data collection nationally to allow cross-centre audit of outcomes and have made the data collection form and joint database available online [11]. 

## Methods

The authors of this study did not consult with their institutional review board (IRB), as the project was a service evaluation and therefore classed as audit. Audits do not require specific permission from IRBs in the UK. Guidance on decision making can be found at the National Research Ethics Service (NRES) website [12] and the Medical Research Council (MRC) Health Research Authority decision tool for audit versus research [13].

### Patients

All patients who underwent EVT for acute ischaemic stroke at the University Hospital of North Staffordshire (UHNS), a tertiary referral centre admitting 1,200 acute strokes per annum, were entered prospectively into the Stoke thrombectomy register. EVT for acute stroke was defined as a procedure undertaken to remove a thrombus from the cerebral circulation. Unless there were intra-procedural reasons to choose a different therapeutic approach, EVT was by mechanical thrombectomy. Any patient in whom the intervention was started (i.e. anaesthesia was commenced) was included, even if the procedure could not be performed or became no longer necessary. 

### The UHNS Endovascular Intervention Protocol

**Figure 1:**
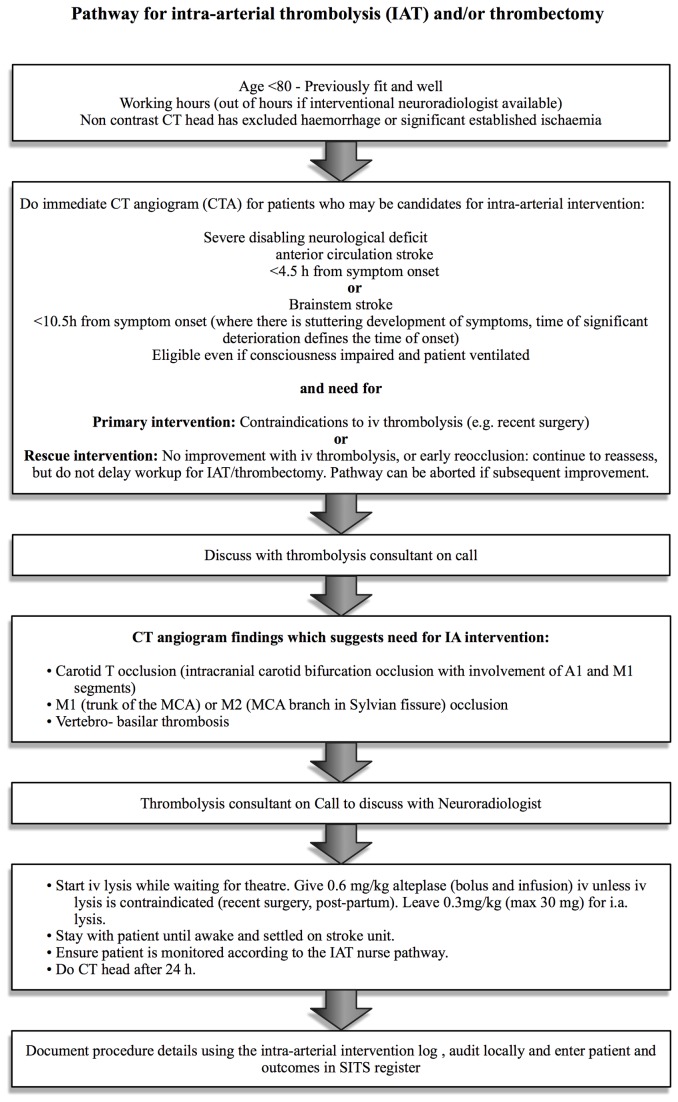
Pathway for endovascular treatment. Treatment pathway used in this study for intra-arterial thrombolysis and/or thrombectomy.

Patients with acute ischaemic stroke are thrombolysed with alteplase unless there are contraindications. A CT angiogram is performed in patients presenting within 9 h of symptom onset who have no contraindications to intravenous contrast. Mechanical thrombectomy is considered in patients under the age of 80 who were previously fit and well and who have an occlusion of a treatable intracranial artery (see Figure 1 for detailed indications). Patients should be within 4.5 h of onset of anterior circulation symptoms and within 10.5 h of posterior circulation symptoms (to allow for recanalization within 6 and 12 hours respectively). We also consider patients above the age of 80 years on an individual basis. In patients with posterior circulation strokes and stuttering development of symptoms the time of onset is defined as the time of significant deterioration (quadriparesis, loss of airway), even if other symptoms (e.g. hemianaesthesia, vertigo, mild ataxia) have started earlier and persisted. Bridging IVT with alteplase (0.9 mg/kg) is given as soon as eligibility is confirmed as a 10% bolus and 90% infusion up to 6 hours after symptom onset for major vessel occlusion in the anterior circulation and up to 12 hours after basilar artery occlusion. Thirty per cent of the infusion is retained for intra-arterial use. If delays are encountered in transfer for the procedure, then the full dose is given intravenously. Bridging ensures that patients receiving EVT are not denied standard treatment of intravenous alteplase. 

The neurointerventional team is alerted as soon as occlusion of an intracranial artery is identified. Preparations for the intervention are halted if symptoms of the stroke resolve. The procedure is performed under general anaesthesia or sedation by one of two interventional neuroradiologists (SN or CJ). Thrombectomy via femoral artery access is attempted using a stent-based thrombus retrieval device. Suction by hand aspiration or using the Penumbra Aspiration Pump (Penumbra Inc., Alameda, CA, USA) is used in more proximal lesions. The remainder of the full dose of alteplase is used for IAT, during, after, or instead of thrombectomy, depending on the requirements of each case. For residual distal thrombus, clot disruption using a microwire may be attempted. Angioplasty and/or stenting are only performed when access to the occluded artery cannot be gained or if there is inadequate blood flow after successful thrombectomy due to a severe proximal stenosis. Intra-arterial nimodipine is given for vasospasm when necessary. 

The procedure is continued until normal arterial flow has been achieved, the interventional team feel no further intervention is possible, or the time limits (6 hours for anterior circulation and 12 hours for posterior circulation) have been reached. In cases where transient reperfusion has been achieved, longer time limits are accepted. The time of the catheter angiogram after final recanalization or termination of the intervention defines the end of the procedure. The current revised protocol is to remove the femoral artery catheter immediately and use a vascular closure device (e.g. StarClose™, Abbott Vascular, Illinois, USA). Admission to the Intensive Care Unit is considered in patients who were ventilated before the procedure and need continued ventilation during recovery and in rare cases where extubation proves difficult. Otherwise, patients are extubated and managed on the acute stroke unit. Blood pressure is maintained at ≤ 185/110 mmHg by labetalol injections and/or infusion of glyceryl trinitrate. Except for the heparin in the solution used to flush out the access catheter, antiplatelet agents and anticoagulants are not used during the first 24 h after thrombolysis, unless a stent has been placed and aspirin 500 mg IV is given during the intervention. If there is no evidence of intracerebral haemorrhage on the CT brain scan after 24 hours, aspirin is started at a dose of 300 mg/day, and replaced by clopidogrel 75 mg/day after 14 days as per UK stroke guidelines [2]. In thrombolysed patients with minor haemorrhagic transformation within the infarct aspirin is started at a dose of 75 mg/day. If a stent has been placed aspirin is given at a dose of 75 mg/day for the first 3 months combined with clopidogrel 75 mg/day to be continued long-term. 

### Data collection

A standard data entry form was used to log data from each procedure. Data recorded include the National Institutes for Health Stroke Scale (NIHSS) [14] with a score of 35 used for unconscious and ventilated patients and 42 for patients who are no longer alive at the time of assessment, the Thrombolysis In Myocardial Infarction (TIMI) score (0 no recanalization of the primary occlusive lesion; I minimal recanalization; II incomplete or partial recanalization; III complete recanalization) [15], and the TICI score (0: no perfusion or ante grade flow; 1 penetration with minimal perfusion; 2a: partial reperfusion in <50% of the vascular territory; 2b: partial reperfusion in >50% of the vascular territory; 3 complete perfusion) [16]. Symptomatic intracranial haemorrhage (sICH) is reported using the Cochrane randomized controlled trial definition, either symptomatic (i.e. associated with a deterioration in the patient’s neurological state), or fatal (i.e. leading directly to death and occurring within the first ten days) [17,18]. This definition includes haemorrhagic transformation of the infarct, haemorrhage elsewhere in the brain, and haemorrhages into the spaces surrounding the brain. As contrast extravasation and haemorrhages cannot be distinguished reliably on plain CT head scan, it also includes contrast extravasation. Neurological deterioration is defined as an NIHSS increase ≥4 from the lowest NIHSS in the first 24 hours. To allow comparisons with Safe Implementation of Treatments in Stroke (SITS) register data we also report sICH using the SITS definition (parenchymatous haemorrhage type 2 (PH2) e.g. haemorrhage in >30% of the infarcted area with significant space-occupation at 22-36 h post-treatment combined with neurological deterioration of ≥4 points from the lowest NIHSS within 24 hours) [18]. Symptomatic cerebral oedema (sCOED) is defined as worsening of NIHSS ≥4 points and brain swelling with midline shift (COED3) within 24-36 h [18]. The modified Rankin Scale (mRS) [19] is assessed at 90 days by telephone or in person by a member of the research team not involved in the intervention to establish functional outcome. Complications at 90 days are assessed in the follow-up clinic and from the patient’s medical records. 

## Results

### Sample and clinical characteristics

A total of 106 patients were treated between 2/12/2009 and 09/01/2013. Three patients were prepared for thrombectomy, but recovered prior to intervention after IVT alone and were not included in the register. Baseline clinical characteristics of the patients included are shown in Table 1. Most of the patients suffered very severe strokes (median NIHSS 18 (IQR 13 - 23) before the intervention; 63% had a total anterior circulation syndrome; 8% had a brain stem stroke, were unconscious, and required ventilation before the procedure. Severity was also reflected in the high proportion of carotid occlusions - common (CCA), internal (ICA), and carotid T - (29%) and basilar artery occlusions (15%). 

**Table 1:** Baseline characteristics of patients.

*Demographic details*	
Age [mean SD] years	64 (14)
Gender [n (%)] male	64 (60)
*Risk factors*	
Hypertension [n (%)]	51 (48)
Atrial fibrillation [n (%)]	35 (33)
Hyperlipidaemia [n (%)]	30 (28)
Smoking [n (%)]	25 (24)
Ischaemic heart disease [n (%)]	26 (25)
Diabetes mellitus [n (%)]	17 (16)
Transient ischaemic attack [n (%)]	8 (8)
Stroke [n (%)]	7 (7)
Chronic kidney disease [n (%)]	5 (5)
Patent foramen ovale [n (%)]	4 (4)
*Clinical findings*	
NIHSS on admission [median (IQR)]	16 (11-21)
NIHSS just before thrombectomy [median (IQR)]	18 (13-23)
Total anterior circulation syndrome [n (%)]	67 (63)
Partial anterior circulation syndrome [n (%)]	15 (14)
Posterior circulation syndrome [n (%)]	23 (22)
*Localization of the most proximal occlusion on catheter angiogram*	
Common carotid artery [n (%)]	3 (3)
Internal carotid artery [n (%)]	13 (12)
Carotid T [n (%)]	15 (14)
Middle cerebral artery M1 [n (%)]	44 (42)*
Middle cerebral artery M2 [n (%)]	5 (5)
Middle cerebral artery M3 [n (%)]	2 (2)
Anterior cerebral artery [n (%)]	0 (0)
Basilar artery [n (%)]	16 (15)
Posterior cerebral artery P1 [n (%)]	1 (1)
Posterior cerebral artery P3 [n (%)]	1 (1)
Vertebral artery [n (%)]	4 (4)
No occlusion [n (%)]	2 (2)

NIHSS: National Institutes for Health Stroke Scale, IQR: interquartile range. *Includes one case of M1 occlusion on CT angiogram where a catheter angiogram could not be done.

### Timelines

Timings of each step in the pathway are given in Table 2. The median time from stroke onset to the end of the procedure (final catheter angiogram) was 6 h 03 min (range 3 h 20 min -15h 20 min). In posterior circulation procedures it was longer than in anterior circulation procedures (8 h 01 min vs. 5 h 55 min) reflecting the wider time frame for eligibility and the more stuttering onset of brain stem strokes. While the median time taken for the endovascular procedure was relatively short (1 h 18 min) it was largely determined by the vascular anatomy of the patient, ranging from 12 min in a 69 year old with a cardioembolic stroke to 4 h 30 min in a 90 year old with basilar occlusion and widespread vascular disease. The overall time from hospital arrival to the end of the procedure was much longer (median 4 h 04 min) due to delays in assembly of the interventional team, travel of staff members into hospital out of hours, and competing demands on the interventional suite and anaesthetic team. 

**Table 2:** Timings for steps in the EVT pathway.

*Individual steps*	Median (h:min)	Interquartile Range (h:min)
Time from stroke onset to hospital admission	1:32	1:00-3:05
Time from admission to start of IV thrombolysis	1:01	0:46-1:35
Time from thrombolysis to neurointerventional lab	0:39	0:22-1:10
Time from lab entry to anaesthesia	0:15	0:10-0:25
Time from anaesthesia to start of procedure (femoral artery puncture)	0:17	0:10-0:25
Time from femoral artery puncture to end of procedure (final catheter angiogram)	1:18	0:58-2:05
Time from final catheter angiogram to leaving the interventional lab	0:20	0:15-0:33
Time from leaving the interventional lab to arrival on the stroke unit	1:13	0:50-1:50
*Composite timings*		
Time from onset to IVT	2:45	2:02-3:36
Time onset to IV thrombolysis (anterior circulation)	2:31	2:00-3:15
Time onset to IV thrombolysis (posterior circulation)	3:58	2:56-6:15
Time from onset to CT angiogram	2:12	1:33-3:33
Time from onset to end of procedure	6:03	5:07-7:02
Time onset to end of procedure (anterior circulation)	5:55	4:51-6:42
Time onset to end of procedure (posterior circulation)	8:01	6:00-10:32
Time from door to end of procedure	4:04	3:04-5:03

Hospital admission is admission to the University Hospital of North Staffordshire (UHNS). This includes some patients who were transferred from other hospitals. Timings do not fully add up since some patients did not have IVT and some had IVT in another hospital before transfer to UHNS.

### Interventions

Interventions were performed under general anaesthesia in 92 (87%) cases. Eighty-eight (83%) cases were treated with mechanical thrombectomy. Devices used for thrombectomy were: Solitaire AB/FR™ (Ev3, Covidien, Mass, USA) (n=63), Acandis APERIO (Pforzheim, Germany) (n=20), Revive SE™ (Codman & Shurtleff Inc. Mass. USA) (n=14), pRESET (Phenox, Bochum, Germany) (n=1), and aspiration via a distal access catheter (n=13); in 2 cases the Penumbra pump was utilized. More than one type of device was used in 18 interventions.

IAT without thrombectomy was performed in 14 (13%) cases, usually when the only occluded vessel was more distal (e.g. P2, P3 M2, M3). 

In four cases (4%) it was impossible to gain access to the carotid artery and perform EVT because of severe atherosclerosis of the aortic arch, tortuosity of the aorta, and, in one case, a large retrosternal goitre. In 2 (2%) patients the vessel (M1 and basilar tip respectively) had recanalized with IVT alone at the time of the catheter angiogram and no visible occlusion remained. In these cases the retained alteplase was given IA. 

Bridging thrombolysis was performed in 86 (81%) of cases. 20 (19%) required angioplasty and/or stenting, 16 for the internal carotid artery (3 angioplasties alone, 2 stents alone, 11 combined), 2 for the middle cerebral artery (both angioplasties, one combined with carotid angioplasty), and 3 for the vertebral artery (1 angioplasty alone, 2 stents alone). Three of the carotid stents were placed to treat pre-existing dissection, the remainder of the angioplasties and stents were required to treat severe flow-limiting stenosis or occlusion and to facilitate access to the thrombus and maintain adequate downstream blood flow in patients with vessel occlusion or critical stenosis. 

### Outcomes and complications

Outcomes and complications are shown in detail in Table 3. Partial or full reperfusion (TICI 2b or 3) flow was achieved in 89 (84%), with full reperfusion (TICI 3) in 58 (55%). This was achieved equally in the proximal anterior and posterior circulation, but in MCA M2+ lesions TICI 2b/3 was only achieved in 57% in keeping with lower rates of MT in these vessels (43%).

**Table 3:** Outcomes and complications.

Site of occlusion n (%)	All sites 106 (100)	CCA/ICA 16 (15)	Carotid T 15 (14)	MCA/M1 44 (52)	MCA/M2+7 (7)	VA/BA/PCA 22 (21)	None 2 (2)
*Recanalization*							
Partial/full reperfusion (TICI 2b or 3)	89 (84)	13 (81)	12 (80)	38 (86)	4 (57)	20 (91)	2 (100)
Partial/full recanalization (TIMI 2 or 3) [n (%)]	94 (89)	14 (88)	13 (87)	39 (89)	6 (86)	20 (91)	2 (100)
***Outcomes at 1 week***							
Change in NIHSS [median]	-10	1	-10	-9	-6	-15	-6
Mortality [n (%)]	6 (6)	2 (13)	3 (20)	1 (2)	0 (0)	0 (0)	0 (0)
*Outcomes by 90 days*							
MRS score ≤ 2 [n (%)]	51 (48)	5 (31)	6 (40)	22 (50)	2 (29)	14 (64)	2 (100)
MRS score ≤ 3 [n (%)]	62 (58)	8 (50)	7 (47)	26 (59)	5 (71)	14 (64)	2 (100)
Mortality [n (%)]	16 (15)	4 (25)	5 (33)	4 (9)	0 (0)	3 (14)	0 (0)
*Procedure-related complications*							
Vasospasm in the ICA [n (%)]	10 (9)	1 (6)	2 (13)	7 (16)	0 (0)	0 (0)	0 (0)
Vasospasm in other vessels (MCA, BA, VA) [n (%)]	9 (8)	1 (6)	1 (7)	5 (11)	0 (0)	2 (9)	0 (0)
Distal clot propagation [n (%)]	8 (8)	0 (0)	0 (0)	8 (18)	0 (0)	0 (0)	0 (0)
Dissection of the access vessel [n (%)]	4 (4)						
Dissection of the target vessel [n (%)]	5 (5)*	1 (6)	1 (7)	2 (5)	1 (14)	0 (0)	0 (0)
Subarachnoid haemorrhage [n (%)]	6 (6)†	2 (13)	1 (7)	2 (5)	1 (14)	0 (0)	0 (0)
Device malfunction [n (%)]	3 (3)‡	1 (6)	1 (7)	0 (0)	1 (14)	0 (0)	0 (0)
Transient contrast leak without SAH [n (%)]	1 (1)	0 (0)	0 (0)	0 (0)	0 (0)	1 (5)	0 (0)
*Complications within the first week after the procedure*							
aICH [n (%)]	27 (25)	4 (25)	4 (27)	15 (34)	0 (0)	4 (18)	0 (0)
sICH [n (%)]	10 (9)§	5 (31)	4 (27)	1 (2)	1 (14)	0 (0)	0 (0)
Cerebral oedema with midline shift [n (%)]	15 (14)	6 (40)	5 (33)	3 (7)	1 (14)	0 (0)	0 (0)
Transient asymptomatic contrast nephropathy [n (%)]	2 (2)	0 (0)	0 (0)	2 (5)	0 (0)	0 (0)	0 (0)
Groin haematoma requiring treatment [n (%)]	0 (0)	0 (0)	0 (0)	0 (0)	0 (0)	0 (0)	0 (0)
*Complications within 90 days*							
Deep vein thrombosis [n (%)]	7 (7)	1 (6)	1 (7)	3 (7)	1 (14)	0 (0)	0 (0)
Pulmonary embolism [n (%)]	4 (4)	0 (0)	0 (0)	2 (5)	1 (14)	0 (0)	0 (0)

*All 5 patients with dissections of the target vessel also had subarachnoid haemorrhage (SAH) (in 5 this was still visible at 24 h and in one it as no longer identifiable at the 24 h CT head scan. †One patient had appearances of a minor subarachnoid bleed at 24 h but no indication of dissection during the procedure. ‡All three were unintended stent detachments (Solitaire FR). §Symptomatic and asymptomatic intracranial haemorrhage (sICH/aICH) is reported using the Cochrane randomized controlled trial definition (either symptomatic (i.e. associated with a deterioration in the patient’s neurological state), or fatal (i.e. leading directly to death), and occurring within the first 10 days). Routine follow-up for patients in our series was at 7 days and 90 days; events after 7 days were checked at the second follow-up. There were no additional haemorrhages between day 7 and day 10. This includes haemorrhagic transformation of the infarct, haemorrhage elsewhere in the brain, and haemorrhages into the spaces surrounding the brain. TIMI: Thrombolysis In Myocardial Infarction score. TICI: Thrombolysis In Cerebral Infarction score. NIHSS: National Institutes of Health Stroke Scale. CCA: common carotid artery, ICA: internal carotid artery, BA: basilar artery, MCA: middle cerebral artery, VA: vertebral artery, PCA: posterior cerebral artery.

The median NIHSS was 18 before the procedure and 8 at 1 week. At 3 months, 51 (48%) of cases achieved functional independence with a mRS score ≤ 2. The overall mortality was 16 (15%) at 3 months. Seven patients died after unsuccessful recanalization, 3 of cerebral oedema, 3 of later complications of the persistent severe stroke, and one of recurrent stroke in the other hemisphere. Nine recanalized, but died of cerebral oedema (6, two of these caused by reocclusion of the treated artery) or pneumonia (3). 

Seventeen (16%) had no or minimal reperfusion (TICI 0 =8, TICI 1=4, TICI 2a =5). Of these, 41% (n=7) died and none of the survivors regained independence. By contrast, in the 89 patients with good reperfusion (TICI 2b or 3), 55% (n=49) made a good recovery, and 10% (n=9) died. 

Significant procedure-related complications included distal emboli (8%), dissection with subarachnoid haemorrhage (5%), and inadvertent stent detachment (3%, all with the Solitaire FR device). There was evidence of intracranial haemorrhage on the CT head scan in 38 (36%) cases, but haemorrhage was symptomatic in only 10 (9%) by the RCT definition. Symptomatic intracerebral haemorrhage (SITS definition) occurred in 2 (2%). Severe cerebral oedema with midline shift (COED3) occurred in 14% of cases with 8% resulting in clinical worsening. There were only minor asymptomatic puncture site haematomas. None required analgesia, transfusion, or surgery. Two patients (2%) had transient decline of renal function attributed to contrast-induced nephropathy which improved without the need for dialysis. By 90 days after the procedure symptomatic deep vein thrombosis and pulmonary embolism were found in 7% and 4% respectively.

### Length of stay and discharge destination

Details of length of stay and discharge disposition are given in Table 4. The median (interquartile range) length of stay was 14 (4-81) days. Eighty-three patients (91% of live discharges) were discharged back home, 33 (31%) within the first week. Of the 15 (14%) patients who required intensive care after the procedure 8 had basilar artery occlusions. Seven out of the 8 patients with basilar artery occlusions were ventilated before the procedure.

**Table 4:** Data relating to service provision.

Patients discharged to care homes (n, % of live discharges) *	8 (9)
Patients discharged to own home/ family (n, % of live discharges) †	83 (91)
Patients discharged home within 7 days (n, % of all patients)	33 (31)
Patients who required intensive care admission (n, % of all patients)	15 (14)
Length of stay in the Intensive Care Unit (ITU) [median, IQR] days‡	3 (1-8)
Length of stay in the Acute Stroke Service [median, IQR] days	7 (4-14)
Length of stay in Inpatient Rehabilitation [median, IQR] days	0 (0-49)
Total length of stay to final discharge from hospital [median, IQR] days§	14 (4-81)

IQR: interquartile range *91 patients were discharged alive to the community, † one died before 90 days. ‡For those admitted to ITU §Includes time in ITU.

## Discussion

Our registry data show that EVT for acute stroke can be done safely and effectively within a hyperacute stroke service in the UK with a reperfusion (TICI ≥2b) rate of 84%, good recovery (mRS≤ 2) in 48% and a low mortality of 15% at 90 days. These outcomes compare favourably to published studies as summarized in a recent systematic review showing recanalization rates between 25% to 96%, mortality 17% (mean, range 7 to 45%), and 90 day mRS ≤2 in 39% (mean, range 15 to 54%) [20].

Recently three RCTs (IMS-3 [5], MRRESCUE [6], and SYNTHESIS [7]) of EVT for acute ischaemic stroke have been published showing no improvement in mortality and disability with intervention. Revascularization rates were low with TICI ≥2a in 76% in IMS-3, 70% in MRRESCUE and unavailable in SYNTHESIS. Most of the earlier, non-randomized studies [20] used more stringent criteria for recanalization e.g. TICI ≥2b or TIMI≥2. In IMS-3 only 41% achieved this level of recanalization, and no data published for the other two studies. Less than half in the active study arms in IMS-3 (44%) and SYNTHESIS (31%) were treated with MT, with IAT used in the remainder. This, and the use of older/less effective devices are likely reasons for lower recanalization rates than in published case studies. 

A significant proportion of recanalized patients do not make a good recovery, a problem described as ‘futile recanalization’. MRRESCUE randomized patients with occlusions in the anterior circulation up to 8h from symptom onset. This very wide time window would explain the low rate of independent survivors (20%) and the lack of effect. Time is clearly a key factor determining whether successful recanalization is associated with better recovery [21]. The RECANALISE study has recently shown that 93% of patients who recanalized within 3 h 30 min were independent at 3 months, compared to only 37% of those recanalized in excess of 4 h 20 min [22]. In our case series the median time from onset to the end of the procedure was 6 h 03 min, with a median door to end of procedure time of 4 h 04 min. Faster in-hospital processes are likely to improve outcomes further. However, time to treatment is only one of several determinants of outcome. In a case series of 623 patients who received EVT, Galimanis et al identified good collaterals, lower NIHSS on admission, and younger age in addition to recanalization and, when collaterals were excluded, time to treatment as predictors of favourable outcome [23]. 

In a comparison with historical controls treated with IVT the RECANALISE study showed a relative risk of 1.2 for a better outcome (mRS≥2) for EVT [22]. We compared our EVT outcomes with those reported for patients of similar age and stroke severity in the SITS register. Using the cut-off date of 1 April 2013, the age range of 22-76, and an NIHSS range of 14-35, we identified a subgroup of 14,145 patients with a mean age of 64 years and a median baseline NIHSS of 18 matching our patient characteristics. In this group of patients treated by IVT 35% were independent, and 19% had died at 90 days. With 48% independent and 15% dead at 90 days, the results of our patients treated with EVT compare favourably with the SITS population treated with IVT alone with a relative risk for a good outcome of 1.4 [24]. The median time from onset to thrombolysis was at 2 h 21 min in the SITS cohort comparable to the 2 h 45 min (where administered) in our cohort. A significant proportion of patients in our cohort were ineligible for thrombolysis and were not thrombolysed (19%) or had bridging thrombolysis late (9% at 279-800 minutes post symptom onset) while preparing for interventional treatment, at a time when thrombolysis is no longer licensed for therapeutic use in acute ischaemic stroke (Table 5). These would be expected to have a worse prognosis with standard care than the SITS cohort. 

**Table 5:** Outcomes for patients treated with late intravenous thrombolysis or with endovascular treatment alone.

	Anterior circulation strokes given IVT after 270 min	Posterior circulation strokes given IVT after 270 min	Patients treated with EVT alone (ineligible for IVT)
Number (%) of cases	5 (5)	5 (5)	20 (19)
Anterior circulation [n (%)]	5 (100)	0 (0)	16 (80)
Posterior circulation [n (%)]	0 (0)	5 (100)	4 (20)
IVT given [n (%)]	5 (100)	5 (100)	0 (0)
Median time onset to IVT [h:min (IQR)]	5:18 (4:50-5:45)	11:00 (9:25-12:21)	N/A
Median time onset to CTA [h:min (IQR)]	4:02 (3:54-5:43)	9:48 (8:27-12:02)	2:25 (2:10-4:39)
Median time onset to start of EVT [h:min (IQR)]	5:49 (5:47-7:31)	10:40 (9:19-12:16)	4:44 (3:55-6:03)
Median time to end of EVT [h:min (IQR)]	7:45 (7:00-8:00)	12:58 (12:00-14:41)	6:07 (5:30-8:55)
TICI 2b/3 flow [n (%)]	5 (100)	5 (100)	13 (65)
MRS≤2 at 90 days [n (%)]	2 (40)	4 (80)	7 (35)

IVT: intravenous thrombolysis. EVT: endovascular treatment. CTA: computerized tomography angiogram. IQR: interquartile range. TICI: Thrombolysis in Cerebral Infarction score. MRS: modified Rankin scale.

Our results showed a high rate of intracranial haemorrhage (36% including intracerebral haemorrhage, SAH, and intraventricular haemorrhage), comparable to IMS-3 (34%, only intracerebral haemorrhages included), but lower than MRRESCUE (58%). A recent review of open studies shows a mean rate of intracerebral haemorrhage of 11% (range 0-45%) [20]. The great majority of haemorrhages in our cohort were non-confluent haemorrhagic transformation, usually in the basal ganglia with no clinical signs. The appearances of haemorrhagic transformation might be due to contrast staining rather than true haemorrhage. It has previously been shown that haemorrhagic/contrast staining areas (HCAs) are common after mechanical thrombectomy, but do not carry an increased risk of symptomatic haemorrhage or poor outcome [25]. Haemorrhages associated with clinical worsening are considerably less common (9%), and in the great majority of these the clinical deterioration was due to coexistent cerebral oedema rather than the haemorrhage itself. Consequently there was a much lower rate of symptomatic intracerebral haemorrhage as defined by SITS, which only counts space occupying parenchymal haemorrhages and restricts this to the first 36 h. We only had one asymptomatic intracerebral haemorrhage (aICH) after 36 hours. These results suggest that our policy of starting low dose antiplatelet agents within 24 h of the intervention in patients with minor haemorrhagic transformation is safe. 

As in most of the recent case series (20) and the IMS-3 and MRRESCUE studies we allowed bridging lysis. This meant that no patient was denied evidence-based, guideline concordant treatment. We also recognize that organizing a thrombectomy takes longer than administration of intravenous thrombolysis. Such delays were seen in SYNTHESIS, where the endovascular group was treated considerably later than the non endovascular group. In addition to allowing faster initiation of treatment, it can be hypothesized that bridging IVT may augment the effectiveness of MT by changing the structure of the thrombus and dissolving small distal emboli not amenable to MT. Our case series shows that MT in the presence of therapeutic doses of alteplase is safe, confirming safety data from IMS-3 and SYNTHESIS and case series [26], and extends these data by showing good outcomes even with late bridging lysis (Table 5).

## Conclusion

Results of thrombectomy with bridging IVT in our service are at least as good as would be expected with IVT alone with no increase in overall risk of death or symptomatic intracerebral haemorrhage. Comparisons with a matched SITS cohort suggest a relative risk of 1.4 for a better outcome than thrombolysis alone. Our data compare favourably with the outcomes of more recent trials involving stent-based devices. These results should be confirmed in well-designed RCTs. However, while this is awaited, and especially in patients where thrombolysis is contraindicated, our data suggest that MT is safe. Until there is clear evidence for benefit, all treated patients should be included in a registry. This could be via SITS International for international comparisons and/or by joining the Stoke Collaborative Thrombectomy Register for more detailed data collection. 
